# Acetylcholine Neuromodulation in Normal and Abnormal Learning and Memory: Vigilance Control in Waking, Sleep, Autism, Amnesia and Alzheimer’s Disease

**DOI:** 10.3389/fncir.2017.00082

**Published:** 2017-11-02

**Authors:** Stephen Grossberg

**Affiliations:** Center for Adaptive Systems, Graduate Program in Cognitive and Neural Systems, Departments of Mathematics & Statistics, Psychological & Brain Sciences and Biomedical Engineering, Boston University, Boston, MA, United States

**Keywords:** acetylcholine, adaptive resonance theory, vigilance, autism, medial temporal amnesia, slow wave sleep, Alzheimer’s disease, nucleus basalis of Meynert

## Abstract

Adaptive Resonance Theory, or ART, is a neural model that explains how normal and abnormal brains may learn to categorize and recognize objects and events in a changing world, and how these learned categories may be remembered for a long time. This article uses ART to propose and unify the explanation of diverse data about normal and abnormal modulation of learning and memory by acetylcholine (ACh). In ART, *vigilance control* determines whether learned categories will be general and abstract, or specific and concrete. ART models how vigilance may be regulated by ACh release in layer 5 neocortical cells by influencing after-hyperpolarization (AHP) currents. This *phasic* ACh release is mediated by cells in the nucleus basalis (NB) of Meynert that are activated by unexpected events. The article additionally discusses data about ACh-mediated *tonic* control of vigilance. ART proposes that there are often dynamic breakdowns of tonic control in mental disorders such as autism, where vigilance remains high, and medial temporal amnesia, where vigilance remains low. Tonic control also occurs during sleep-wake cycles. Properties of Up and Down states during slow wave sleep arise in ACh-modulated laminar cortical ART circuits that carry out processes in awake individuals of contrast normalization, attentional modulation, decision-making, activity-dependent habituation, and mismatch-mediated reset. These slow wave sleep circuits interact with circuits that control circadian rhythms and memory consolidation. Tonic control properties also clarify how Alzheimer’s disease symptoms follow from a massive structural degeneration that includes undermining vigilance control by ACh in cortical layers 3 and 5. Sleep disruptions before and during Alzheimer’s disease, and how they contribute to a vicious cycle of plaque formation in layers 3 and 5, are also clarified from this perspective.

## 1 Introduction: Modeling Neural Systems that Include Neuromodulators

*Wikipedia* states that “Neuromodulation is the physiological process by which a given neuron uses one or more chemicals to regulate diverse populations of neurons. This is in contrast to classical synaptic transmission, in which one presynaptic neuron directly influences a single postsynaptic partner…” Thus, in order to understand how a neuromodulator works, one needs to characterize the neural system or systems upon which it acts, notably the neural mechanisms of which it is composed, the psychological functions that it enables, and how the neuromodulator may alter both. A complete understanding of a neuromodulator thus requires that a linkage be established between brain mechanisms and mental functions. Without such a link, the mechanisms of the brain have no functional significance, the functions of behavior have no mechanistic explanation, and it will remain unclear how neuromodulators do their job.

Establishing such a link with sufficient clarity for it to be scientifically predictive requires rigorous mathematical models that can simultaneously describe multiple levels of brain and behavioral organization. A rapidly growing number of such models can now quantitatively simulate the neurophysiologically recorded dynamics of identified nerve cells in known anatomies and the behaviors that they control. In addition to providing unified explanations of abundant psychological and neurobiological data, many predictions of these models have been supported by subsequent experiments over the years. See Grossberg (2013, 2017) for reviews.

**Figure 1:**
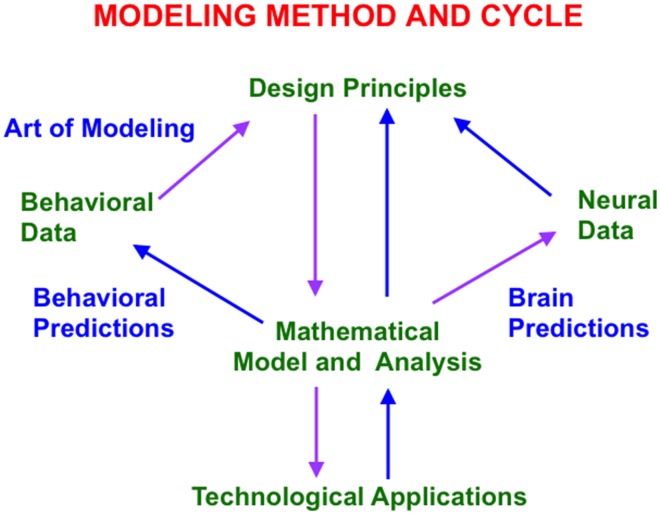
This modeling method and cycle clarifies how increasingly refined neural models can explain and predict increasingly large interdisciplinary behavioral and neurobiological data bases. See text for details.

One successful approach uses a theoretical method that has been developed and applied during the past 60 years (Grossberg, 1999). Because *brain* evolution needs to achieve *behavioral* success, this “method of minimal anatomies” begins with a theoretical analysis of scores or even hundreds of parametrically structured behavioral experiments (Figure 1). Starting with behavioral data enables derivation of models whose brain mechanisms have been shaped during evolution by behavioral success. A unified mechanistic explanation of large numbers of behavioral experiments is sought to rule out many otherwise seemingly plausible answers.

The method uses such a large behavioral database to discover novel design principles and mechanisms to explain how an individual, behaving in real time, can generate these data as emergent properties. Despite being based on psychological constraints, the minimal mathematical models that realize these design principles have always looked like part of a brain (Figure 1). Sixty years of modeling have hereby supported the hypothesis that brains look the way that they do because they embody natural computational designs whereby individuals can autonomously adapt to changing environments in real time. Moreover, this kind of behavior-to-principle-to-model-to-brain theoretical derivation has often disclosed unexpected functional roles of the derived brain mechanisms that are not clear from neural data alone.

The *minimal* model is first derived. Minimality here means that removing any of the model’s mechanisms prevents it from explaining a key set of previously explained data. Once a connection is made top-down from behavior to brain, mathematical and computational analysis discloses what the minimal model, and its variations, can and cannot explain. An analysis of this “boundary between the known and unknown” focuses attention upon design principles that the current model does not embody. These new design principles and their mechanistic realizations are then consistently assimilated into the model. Indeed, if such a model unlumping process, or “embedding”, is not possible, then that is strong meta-theoretical evidence that the current model contains a serious error.

This theoretical cycle has been successfully repeated multiple times since 1957, thereby disclosing a series of progressively unlumped models, each consistent with the others, and each with an increasing broad explanatory and predictive range. Although one cannot “derive an entire brain” in one step using this method, an increasing number of these models can individually explain psychological, neurophysiological, neuroanatomical, biophysical, and even biochemical data. They hereby illustrate a sense in which the classical Mind/Body Problem is currently being solved. As part of this “conceptual evolution” of models, increasingly detailed insights about various roles for neuromodulators have been discovered. Some of these progressively unlumped models are described in this article, along with new results as well, especially about how properties of slow wave sleep and Alzheimer’s disease are clarified by model laminar cortical circuits that also explain many data about normal awake behaviors.

## 2 Category Learning via Feature-Category Resonances

### 2.1 Adaptive Resonance Solves the Stability-Plasticity Dilemma

**Figure 2:**
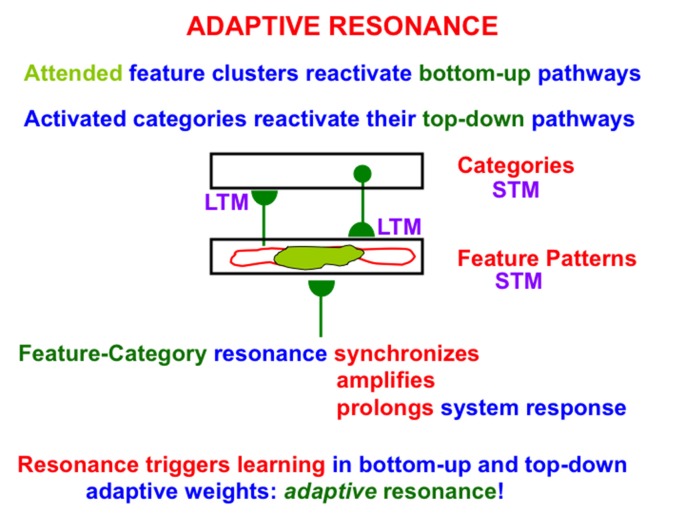
A feature-category resonance can develop when there is a good enough match between a bottom-up input pattern to a network of feature detectors and a learned top-down expectation from an active recognition category. The resonant state synchronizes, amplifies, and prolongs the responses of cells within it, which create and sustain an attentional focus, and thereby triggers learning in the corresponding bottom-up adaptive filters and top-down expectations, hence the name adaptive resonance. At the same time, outliers are suppressed, thereby enabling fast learning without catastrophic forgetting. See text for details.

One such model, called Adaptive Resonance Theory, or ART, proposes how the brain learns categories whereby to attend, recognize, and predict objects and events. This is accomplished in ART using feedback interactions between attended feature patterns and an active recognition category, leading to a *feature-category resonance* (Figure 2). In this conception, attended feature patterns activate bottom-up adaptive filter pathways, while activated recognition categories active their top-down learned expectation pathways.

Bottom-up inputs to the feature pattern level are matched against active top-down expectations. An active top-down expectation, in the absence of bottom-up inputs, can modulate, or prime, the feature-selective cells to anticipate expected feature patterns, but cannot, by themselves, fire these cells. When a good enough match occurs, the system locks into an attentive *resonant state* that synchronizes, amplifies, and prolongs system response (Figure 2). Such a feature-category resonance drives the recognition learning process whereby the adaptive weights, or LTM traces, in the bottom-up and top-down pathways can learn from the patterns of resonating activities, or STM traces; hence the term *adaptive* resonance. By focusing on attended features, the resonance also suppresses unattended features, thereby helping to solve the *stability-plasticity dilemma* (Grossberg, 1980), whereby the brain can learn quickly, without being also forced to catastrophically forget already learned, but still useful, knowledge.

ART has predicted that *all conscious brain states are resonant states*, and that feature-category resonances support the recognition of objects and events (Grossberg, 1980, 2017). More generally, ART predicts a link between brain processes of consciousness, learning, expectation, attention, and synchrony; the so-called CLEARS processes, and that all brain systems that are capable of supporting conscious experiences embody such a linkage between CLEARS processes.

### 2.2 Attentional and Orienting Systems Regulate Category Learning and Search

**Figure 3:**
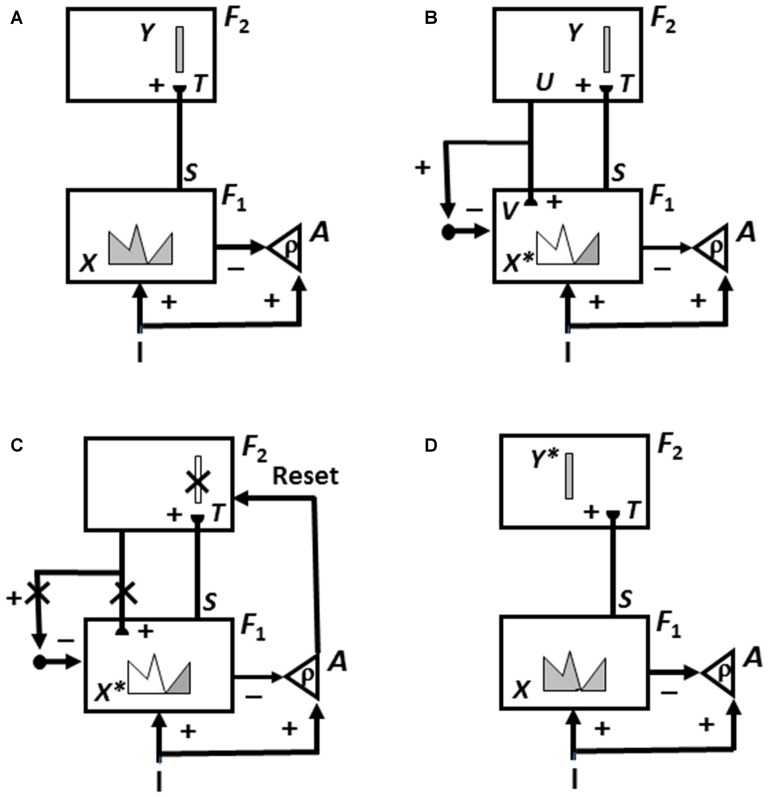
How Adaptive Resonance Theory (ART) searches for and learns a new recognition category using cycles of match-induced resonance and mismatch-induced reset due to interactions of an attentional system and an orienting system. Active cells are shaded gray; inhibited cells are not shaded.** (A)** Input pattern *I* is instated across feature detectors at level *F*_1_ of the attentional system as an activity pattern *X*, at the same time that it generates excitatory signals to the orienting system *A* with a gain *ρ* that is called the *vigilance* parameter. Activity pattern *X* generates inhibitory signals to the orienting system *A* as it generates a bottom-up input pattern *S* to the category level *F*_2_. A dynamic balance within *A* between excitatory inputs from *I* and inhibitory inputs from *S* keeps *A* quiet. The bottom-up signals in *S* are multiplied by learned adaptive weights to form the input pattern *T* to *F*_2_. The inputs *T* are contrast-enhanced and normalized within *F*_2_ by recurrent lateral inhibitory signals that obey the membrane equations of neurophysiology, otherwise called shunting interactions. This competition leads to selection and activation of a small number of cells within *F*_2_ that receive the largest inputs. The chosen cells represent the category Y that codes for the feature pattern at *F*_1_. In this figure, a winner-take-all category is chosen, represented by a single cell (population). **(B)** The category activity *Y* generates top-down signals *U* that are multiplied by adaptive weights to form a prototype, or critical feature pattern, *V* that encodes the expectation that the active *F*_2_ category has learned for what feature pattern to expect at *F*_1_. This top-down expectation input *V* is added at *F*_1_ cells using the ART Matching Rule. If *V* mismatches *I* at *F*_1_, then a new short-term memory (STM) activity pattern X* (the gray pattern), is selected at cells where the patterns match well enough. In other words, X* is active at *I* features that are confirmed by *V*. Mismatched features (white area) are inhibited. When *X* changes to X*, total inhibition decreases from *F*_1_ to *A*. **(C)** If inhibition decreases sufficiently, the orienting system *A r*eleases a nonspecific arousal burst to *F*_2_; that is, “novel events are arousing”. Within the orienting system *A*, a vigilance parameter *ρ* determines how bad a match will be tolerated before a burst of nonspecific arousal is triggered. This arousal burst triggers a memory search for a better-matching category, as follows: Arousal resets *F*_2_ by inhibiting *Y*. **(D)** After *Y* is inhibited, *X* is reinstated and *Y* stays inhibited as *X* activates a different winner-take-all category Y*, at *F*_2_. Search continues until a better matching, or novel, category is selected. When search ends, an attentive resonance triggers learning of the attended data in adaptive weights within both the bottom-up and top-down pathways. As learning stabilizes, inputs *I* can activate their globally best-matching categories directly through the adaptive filter, without activating the orienting system (adapted with permission from Carpenter and Grossberg, 1987).

Category learning in ART is controlled by interactions between an attentional system and an orienting system (Figure 3). These two systems embody computationally *complementary* laws (Grossberg, 2000a). The *attentional system* (levels *F*_1_ and *F*_2_ in Figure 3) carries out processes like attention, category learning, expectation, and resonance in response to familiar and expected events. The *orienting system* (level *A* in Figure 3) enables the attentional system to learn about unfamiliar and unexpected information using processes like reset, memory search, and hypothesis testing. The attentional system includes brain regions like temporal cortex and prefrontal cortex (PFC). The orienting system includes brain regions like the nonspecific thalamus and hippocampus (HPC).

If an input pattern causes a sufficiently bad mismatch to occur within the attentional system, it will activate the orienting system. The orienting system, in turn, resets the active recognition category and initiates a memory search, or hypothesis-testing for a better-matching category, possibly an entirely new one. Figure 3 summarizes this ART search and learning cycle.

Object attention is carried in an ART network by a *top-down, modulatory on-center, off-surround network*. Such a circuit is said to obey the ART Matching Rule. Due to the ART Matching Rule, attention can modulate, sensitize, or prime an expected critical pattern of feature-selective cells, but cannot fully excite them unless enough of them also receive matched bottom-up featural inputs. The off-surround network can actively inhibit unexpected features at the same time. Carpenter and Grossberg (1987) have mathematically proved that category learning is stable in response to an arbitrary sequence of input patterns if the ART Matching Rule is obeyed, but can easily become unstable if it is not, leading to catastrophic forgetting of previously learned categories. See Grossberg (2013, 2017) for a summary of the abundant psychological and neurobiological evidence that supports the prediction that object attention obeys the ART Matching Rule.

### 2.3 Vigilance Control Determines the Criterion for Resonance vs. Reset

How good a match is required for resonance, sustained attention, learning, and consciousness, to occur? This criterion is set by a *vigilance parameter* *ρ* that is computed within the orienting system (Carpenter and Grossberg, 1987). The size of the vigilance parameter determines the generality of the recognition categories that will be learned. If vigilance is high, then learning of a concrete or specific category occurs, such as recognition of a frontal view of a friend’s face. If vigilance is low, then learning of an abstract or general category occurs, such as recognition that everyone has a face. In general, vigilance is chosen as low as possible to conserve memory resources, without causing a reduction in predictive success. Because this baseline vigilance level is initially set at the lowest level that has led to predictive success in the past, ART models try to learn the most general category that is consistent with the data. This tendency can lead to the type of overgeneralization that is seen in young children (Brooks et al., 1999) until subsequent learning leads to category refinement (Tomasello and Herron, 1988).

When a given task requires a finer categorization, vigilance is raised. Vigilance can be automatically adjusted to learn either specific or general information in response to predictive failures, or disconfirmations, within each environment. Such a predictive failure could occur, for example, if a viewer classifies an object as a dog, whereas it is really a fox. Within ART, such a predictive disconfirmation causes a memory search that shifts attention to focus on a different combination of features that can successfully be used to recognize that the object is, in fact, a fox, and perhaps to recognize other foxes as well.

### 2.4 How Vigilance Is Computed

Vigilance is computed within the orienting system of an ART model (Figures 3B–D). This can happen because the orienting system measures how good a match occurs in the attentional system. It does so in the following way: An input pattern I activates two bifurcating pathways. One pathway activates feature detectors at level F_1_ in the attentional system. This activity pattern is denoted by X in Figure 3A. After a top-down expectation V is also activated, the number of active feature detector cells decreases as the match of I with the top-down expectation gets worse. This attended activity pattern is denoted by X* in Figures 3B,C. The second pathway that is activated by I sends converging excitatory signals to the orienting system A. After the feature detectors in F_1_ are activated, they also activate two bifurcating pathways. One pathway sends a signal pattern S via an adaptive filter to level F_2_, where they activate category-coding cells that compete to be stored in short-term memory (STM). The other pathway sends converging inhibitory signals to the orienting system A. In this way, I and X* compete within A. A nonspecific reset signal is emitted by A if the inhibition from X* to A is less than the excitation from I to A.

Here is where the vigilance parameter plays a role. The vigilance parameter *ρ* multiplies the bottom-up excitatory inputs I to A; that is, *ρ* is the *gain* of the excitatory inputs to A. The orienting system A is activated when the total excitatory input *ρ*I is greater than the total inhibition from the features X* across F_1_ that survive top-down matching. This occurs when *ρ*|*I*| − |X*| > 0, where |.| denotes the number of positive inputs or matched features. This inequality can be rewritten as *ρ* > |X*| |*I*|^−1^ to show that the orienting system is activated whenever *ρ* is chosen higher than the ratio of active X* matched features in F_1_ to total features in I. In other words, the vigilance parameter controls how bad a match can be before reset of the current category, and search for a new category, is initiated.

If the vigilance parameter is low, then many exemplars can all influence the learning of a shared category prototype, by chipping away at the features that are not shared with all the coded exemplars. If the vigilance parameter is high, then even a small difference between a new exemplar and a known prototype (e.g., F vs. E) can drive the search for a new category with which to represent F.

### 2.5 Minimax Learning by Match Tracking: Learn the Most General Predictive Categories

Vigilance can be automatically adjusted in response to a predictive disconfirmation (e.g., E is predicted in response to F). One particularly useful way is called *match tracking*. Suppose that on every learning trial, a predictive failure causes vigilance to increase by the smallest amount that can trigger reset of the currently active category. That is, increase *ρ* until *ρ* > |X*||*I*|^−1^. Reset is followed by a memory search for a new recognition category that can correct the error. As a result of such a memory search, a category will be learned that is just general enough to eliminate the error, because every increase in vigilance reduces the generality of learned categories, and vigilance was increased by the minimum amount that was needed to learn a new category. This scheme is called *match tracking* because vigilance tracks the degree of match between the input pattern I and the attended feature pattern X*. Match tracking leads to *minimax learning*, or learning that can *minimize* predictive errors while it conjointly *maximizes* category generality. In other words, match tracking uses the minimum memory resources that are needed to correct the predictive error.

Because vigilance can vary during match tracking in a manner that reflects current predictive success, recognition categories capable of encoding widely differing degrees of generalization or abstraction can be learned by a single ART system (Carpenter and Grossberg, 1987, 1991). Thus a single ART system may be used, say, to learn abstract prototypes with which to recognize abstract categories of faces and dogs, as well as “exemplar prototypes” with which to recognize individual views of faces and dogs, depending on task requirements.

## 3 Two Distinct Processes of Memory Consolidation

ART has predicted that two different, but interacting, memory consolidation processes occur, one during the learning of perceptual or cognitive recognition categories, and the other during the learning of cognitive-emotional interactions.

The consolidation of recognition categories is illustrated by the search and learning cycle that is summarized in Figure 3. As sequences of inputs are practiced over learning trials, the memory search process leads to learning of recognition categories that are stably maintained during subsequent experiences. As categorization of familiar inputs stabilizes, memory search automatically ends and familiar inputs directly access the category whose prototype provides the globally best match, without undergoing any search, while unfamiliar inputs can continue to engage the orienting system to trigger memory searches for better categories until they also become familiar. Direct access to a familiar recognition category represents a kind of dynamically-maintained memory consolidation that can occur entirely within perceptual and cognitive circuits to discover and learn stable recognition categories.

Carpenter and Grossberg (1987) have mathematically proved this property in an ART model. In other words, ART provides a solution of the *local minimum problem* that various other algorithms, such as back propagation (Baldi and Hornik, 1989; Gori and Tesi, 1992), and its Deep Learning refinement (LeCun et al., 2015), do not solve. This process of search and category learning continues until the memory capacity, which can be chosen arbitrarily large, is fully utilized.

ART has predicted that this learning and memory consolidation process often utilizes cortico-hippocampal interactions, where thalamocortical circuits play the role of the attentional system, and the hippocampus playing the role of the orienting system (Figure 3). This conception is supported by several types of experiments. In particular, the role of hippocampus in mismatch-mediated novelty detection has been known for many years (Sokolov, 1960; Vinogradova, 1975; Deadwyler et al., 1979, 1981). Indeed, the hippocampal CA1 and CA3 regions have been shown to be involved in a process of comparison between a prior conditioned stimulus and a current stimulus by rats in a non-spatial auditory task, the continuous non-matching-to-sample task (Sakurai, 1990). During performance of the task, single unit activity was recorded from several areas: CA1 and CA3, dentate gyrus (DG), entorhinal cortex, subicular complex, motor cortex (MC), prefrontal cortex and dorsomedial thalamus. GO and NO-GO responses indicated whether the current tone was perceived, respectively, as the same as (match) or different from (mismatch) the preceding tone. About half of the units in MC, CA1, CA3 and DG had increments of activity immediately prior to a GO response, and were thus implicated in motor or decisional aspects of making a match response. On mismatch trials, units were also found in CA1 and CA3 with activity correlated to a correct NO-GO response. Otto and Eichenbaum (1992) furthermore reported that CA1 cells compare cortical representations of current perceptual processes to previous representations stored in parahippocampal and neocortical structures to detect mismatch in an odor-guided task. They noted that “the hippocampus maintains neither active nor passive memory representations” (p. 332).

Human Event-Related Potential, or ERP, data of Banquet and Grossberg (1987) supported the prediction that, during an ART memory search, sequences of mismatch (Figure 3B), arousal (Figure 3C) and reset (Figure 3D) events occur. These events were interpreted in terms of properties of P120, N200 and P300 ERPs, respectively, which, as predicted, occur together in the predicted temporal order.

More recently, Brincat and Miller (2015) reported neurophysiological data that support the distinction between a category learning attentional system that includes prefrontal cortex, and an orienting system that includes the hippocampus. Their data from prefrontal cortex (PFC) and hippocampus (HPC) in monkeys learning object-pair associations led them to conclude (p. 576): “PFC spiking activity reflected learning in parallel with behavioral performance, while HPC neurons reflected feedback about whether trial-and-error guesses were correct or incorrect…Rapid object associative learning may occur in PFC, while HPC may guide neocortical plasticity by signaling success or failure via oscillatory synchrony in different frequency bands”.

**Figure 4:**
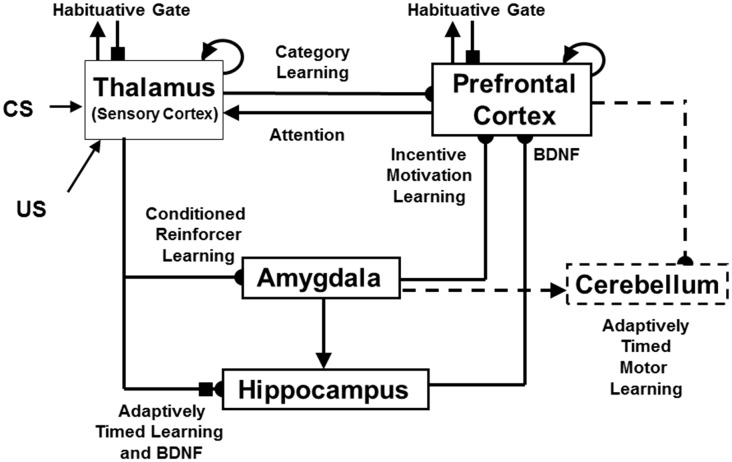
The *neurotrophic START*, or nSTART, macrocircuit is formed from parallel and interconencted networks that support both delay and trace conditioing. Connectivity between thalamus and sensory cortex includes pathways from the amygdala and hippocampus (HPC), as does connectivity between sensory cortex and prefrontal cortex (PFC), specifically orbitofrontal cortex. These circuits are homologous. Hence the model lumps the thalamus and sensory cortex together and simulates only sensory cortical dynamics. Multiple types of learning and neurotrophic mechanisms of memory consolidation cooperate in these circuits to generate adaptively timed responses. Connections from sensory cortex to orbitofrontal cortex support category learning. Reciprocal connections from orbitofrontal cortex to sensory cortex support attention. Habituative transmitter gates modulate excitatory conductances at all processing stages. Connections from sensory cortex to amygdala support conditioned reinforcer learning. Connections from amygdala to orbitofrontal cortex support incentive motivation learning. Hippocampal adaptive timing and BDNF bridge temporal delays between conditioned stimuli (CS) offset and unconditioned stimuli (US) onset during trace conditioning acquisition. BDNF also supports long-term memory consolidation within sensory cortex to hippocampal pathways and from hippocampal to orbitofrontal pathways. The pontine nuclei serve as a final common pathway for reading-out conditioned responses. Cerebellar dynamics are not simulated in nSTART. Key: arrowhead = excitatory synapse; hemidisc = adaptive weight; square = habituative transmitter gate; square followed by a hemidisc = habituative transmitter gate followed by an adaptive weight (reprinted with permission from Franklin and Grossberg, 2017).

A second kind of memory consolidation occurs during cognitive-emotional interactions. During this kind of consolidation, after learning trials end, early vs. late ablations of hippocampus, amygdala, orbitofrontal cortex and thalamus can have different effects on the memory consolidation process. Although this memory consolidation process is not an explanatory target of the current article, mechanistic explanations of this complex data pattern are provided in Franklin and Grossberg (2017), along with computer simulations of the main properties of these consolidation data. These explanations also suggest a role for neuromodulation by describing how brain-derived neurotrophic factor, or BDNF, abets the memory consolidation process. This model is accordingly called the *neurotrophic Spectrally Timed ART*, or nSTART, model due to the simulated role of BDNF in the memory consolidation process (Figure 4).

The process of *Spectral Timing* that is part of the nSTART name models how the hippocampus can bridge temporal gaps, that are often hundreds of milliseconds long, between the stimuli that are being associated through learning. Such temporal gaps between conditioned stimuli (CSs) and unconditioned stimuli (USs) occur during trace conditioning and delayed-non-match-to-sample, among other experimental paradigms. The hippocampus uses Spectral Timing to bridge these temporal gaps in an adaptively timed way that can be tuned to match the interstimulus interval between the stimuli being associated. The nSTART model, and the START model before it (Grossberg and Schmajuk, 1989; Grossberg and Merrill, 1992, 1996; Fiala et al., 1996), posited a critical role for the metabotropic glutamate receptor, or mGLuR, system in defining the spectra that bridge these long temporal gaps. nSTART further proposes how learning of this temporal bridge is modulated by BDNF (Figure 4).

In summary, ART proposes that two types of memory consolidation can occur: One uses cortico-hippocampal interactions to enable category learning to occur, and to achieve direct access, without hippocampal involvement, to familiar recognition categories after they are learned. The other strengthens partially learned recognition categories and other learned associations during cognitive-emotional interactions using the ability of the hippocampus to bridge temporal gaps and to thereby provide modulatory support for the consolidation of these learning processes.

## 4 Acetylcholine Neuromodulation in Vigilance Control

Since the concept of vigilance control was first mathematically described and simulated by Carpenter and Grossberg (1987), quite a bit of new modeling and data have been published that support and refine ART predictions about how vigilance may be regulated in the brain. In particular, neuromodulation by ACh seems to play a major role in vigilance control through brain regions like the nucleus basalis (NB) of Meynert (Grossberg and Versace, 2008; Palma et al., 2012a,b). More will be said about how this is proposed to work in Section 8.

## 5 High or Low Vigilance Influence Autism or Amnesia Symptoms: Vigilance Diseases

It has also been predicted that vigilance cannot dynamically adjust itself sufficiently in some individuals to flexibly respond to task demands, leading to problems of attention, learning, and recognition. Grossberg and Seidman (2006) have, for example, proposed that various individuals with autism have their vigilance stuck at an abnormally high value, leading to the learning of abnormally concrete and hyperspecific recognition categories, as well as to a correspondingly narrow focus of attention. Indeed, the *imbalanced Spectrally Timed ART*, or iSTART, model of Grossberg and Seidman (2006) proposes that *hypervigilance leads to hyperspecific learning*.

Psychophysical experiments have successfully tested the prediction about hyperspecific recognition and attention in high-functioning autistic individuals (Church et al., 2010; Vladusich et al., 2010). It is also known that there is abnormal cholinergic activity in the parietal and frontal cortices of autistic individuals that is correlated with abnormalities in the nucleus basalis (Perry et al., 2001), consistent with the predicted role of the nucleus basalis and ACh in regulating vigilance.

Low vigilance has been predicted to occur in various individuals with medial temporal amnesia. Abnormal ACh modulation could, in principle, cause this problem as well. It remains to more thoroughly study how ACh dynamics may be impaired in autistic and amnesic individuals.

Even if ACh modulation is normal, a hippocampal lesion may damage or eliminate the orienting system during the cortico-hippocampal interactions that control resonance and reset in Figure 3. If mismatch-mediated reset and memory search are prevented, the ability to learn new categories will be degraded. Such a hippocampal lesion that eliminates reset would, in effect, reduce vigilance to zero. Any category learning that can occur without mismatch-mediated reset and memory search would only form very general categories.

Carpenter and Grossberg (1993) and Grossberg (2013) have noted how such a disruption of memory search when the model hippocampus is ablated can qualitatively explain quite a few data about medial temporal amnesia. These properties include unlimited anterograde amnesia because memory search is no longer possible; limited retrograde amnesia because memory search is no longer needed after category memories consolidate and direct access to them occur; and difficulties in orienting to novel cues, perseveration, and a failure of recombinant context-sensitive processing because a mismatch-mediated reset is no longer possible.

Differential learning by amnesics and normal individuals has been reported on easy vs. demanding categorization tasks. Knowlton and Squire (1993) have shown that amnesic subjects and normal subjects perform equally well on easy categorization tasks, but amnesic subjects perform far worse on more demanding tasks. Knowlton and Squire (1993) surmised from their data that two separate memory systems exist. In contrast, Zaki et al. (2003) quantitatively fit the Knowlton and Squire data with a single exemplar-based model whose sensitivity parameter was chosen lower for amnesics than for normal subjects. This exemplar model is usually expressed in terms of formal algebraic equations that implicitly use non-local interactions to compute its most important equations. Amis et al. (2009) have shown that the Zaki et al. (2003) exemplar model may, in fact, be interpreted as a real-time dynamical process undergoing only locally defined interactions. This dynamical process computes learned prototypes of top-down expectations that strikingly resemble ART processes. With this comparison in mind, it can be shown that a low sensitivity parameter *c* in the exemplar model (see their equation (4)) plays a role similar to that played by a low vigilance parameter *ρ* in an ART model that can be caused by ablation of the ART orienting system in Figure 3.

Combining these several types of experimental paradigms described in Sections 2.6–2.8 should enable a deeper mechanistic understanding to be achieved. This will be particularly the case when the predicted role of ACh in vigilance control, as explained in the following sections, is also included.

## 6 Hippocampal Functions: Space, Time, Novelty, Consolidation and Episodic Learning

The ART models that are summarized above explain how more than one hippocampal process may be disrupted by a hippocampal lesion during the memory consolidation process. In particular, hippocampal ablation can interfere with both *novelty-sensitive memory search* that helps to discover and learn better-matching categories, and *adaptively timed maintenance of resonances* that can support learning between stimuli that are separated in time. Both of these processes were included in START model circuit (Grossberg and Schmajuk, 1989; Grossberg and Merrill, 1992, 1996), but without the enhancements that have enabled the nSTART model (Franklin and Grossberg, 2017) to simulate a more complete set of data about how early vs. late lesions of hippocampus, amygdala, orbitofrontal cortex and thalamus alter memory consolidation after delay and trace conditioning.

Franklin and Grossberg (2017) also review other functional roles that are played by the hippocampus *in vivo*, including critical role for the hippocampus in spatial navigation through interactions between entorhinal grid cells and hippocampal place cells. The hippocampus hereby illustrates an issue that is confronted whenever one studies how a given brain region works: Why does each brain region support a particular combination of processes, rather than a different one? How do these processes interact in a way that makes functional sense of the fact that they all take place within the same brain region? Recent modeling articles about how grid and place cells may develop clarify why this is the case; e.g., Grossberg and Pilly (2012, 2014) and Pilly and Grossberg (2012, 2013, 2014) by noting, in particular, how homologous circuits in parallel entorhinal-hippocampal streams may give rise to maintenance of adaptively-timed motivated attention on the one hand (spectral timing) via the lateral entorhinal-hippocampal cortical stream, and spatial navigation using grid cells and place cells on the other (spectral spacing) via the medial entorhinal-hippocampal cortical stream.

## 7 Laminar Neocortical Art Circuits: 3D Laminart, cARTWORD and List Parse

A finer understanding of how vigilance is proposed to work is achieved in ART models that are realized by laminar neocortical circuits with spiking neurons. These and related modeling developments are mentioned here to illustrate the generality of the conclusions below about ACh-modulated vigilance control in laminar neocortical circuits, in both normal and abnormal brains.

**Figure 5:**
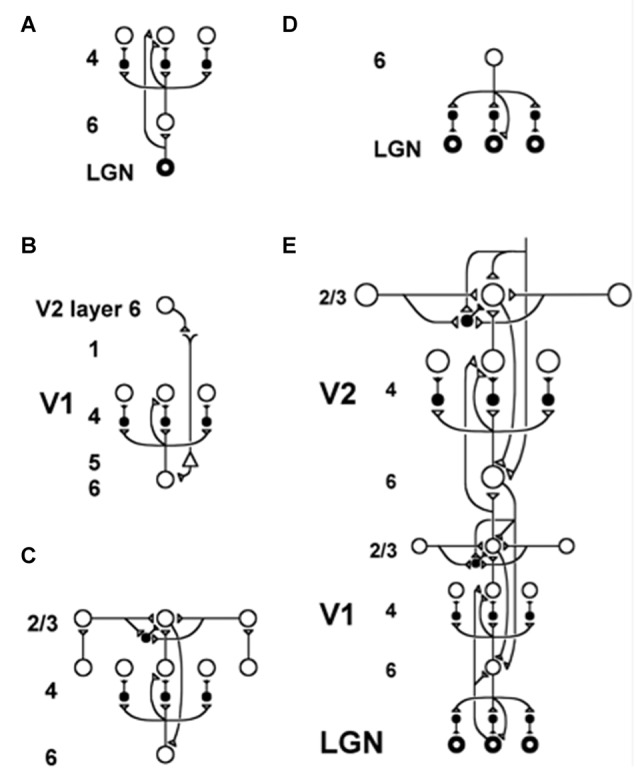
The LAMINART model clarifies how bottom-up, horizontal and top-down interactions within and across cortical layers in V1 and V2 interblob and pale stripe regions, respectively, carry out bottom-up adaptive filtering, horizontal grouping, and top-down attention. Similar interactions seem to occur in all six-layered cortices. Inhibitory interneurons are shown filled-in black. **(A)** The lateral geniculate nucleus (LGN) provides bottom-up activation to layer 4 via two routes. Firstly, it makes a strong connection directly into layer 4. Secondly, LGN axons send collaterals into layer 6, and thereby also activate layer 4 via the 6→4 on-center off-surround path. Thus, the combined effect of the bottom-up LGN pathways is to stimulate layer 4 via an on-center off-surround, which provides divisive contrast normalization (Grossberg, 1973, 1980; Heeger, 1992) of layer 4 cell responses. **(B)** Folded feedback carries attentional signals from higher cortex into layer 4 of V1, via the modulatory 6→4 path. Corticocortical feedback axons tend preferentially to originate in layer 6 of the higher area and to terminate in the lower cortex’s layer 1 (Salin and Bullier, 1995, p.110), where they can excite the apical dendrites of layer 5 pyramidal cells whose axons send collaterals into layer 6 (the triangle in the figure represents such a layer 5 pyramidal cell). Several other routes through which feedback can pass into V1 layer 6 also exist. Having arrived in layer 6, the feedback is then “folded” back up into the feedforward stream by passing through the 6→4 on-center off-surround path (Bullier et al., 1996). Although the off-surround is drawn as a feedforward circuit, for simplicity, the inhibitory interneurons actually inhibit one another as well to form a self-normalizing recurrent circuit. This property is needed to make the circuit work properly, as well as to explain sleep Up and Down state properties, as described in Section 11. **(C)** Connecting the 6→4 on-center off-surround to the layer 2/3 grouping circuit: Like-oriented layer 4 simple cells with opposite contrast polarities compete (not shown) before generating half-wave rectified outputs that converge onto layer 2/3 complex cells in the column above them. Just like attentional signals from higher cortex, groupings that form within layer 2/3 also send activation into the folded feedback path, to enhance their own positions in layer 4 beneath them via the 6→4 on-center, and to suppress input to other groupings via the 6→4 off-surround. The layer 6-to-4 circuit thus realizes a decision interface at which pre-attentive groupings and top-down attention cooperate and compete to determine a consensus decision. There exist direct layer 2/3→6 connections in macaque V1, as well as indirect routes via layer 5. **(D)** Top-down corticogeniculate feedback from V1 layer 6 to LGN also has an on-center off-surround anatomy, similar to the 6→4 path. The on-center feedback selectively enhances LGN cells that are consistent with the activation that they cause (Sillito et al., 1994), and the off-surround contributes to length-sensitive (endstopped) responses that facilitate grouping perpendicular to line ends. **(E)** The entire V1/V2 circuit: V2 repeats the laminar pattern of V1 circuitry, but at a larger spatial scale. In particular, the horizontal layer 2/3 connections have a longer range in V2, allowing above-threshold perceptual groupings between more widely spaced inducing stimuli to form (Amir et al., 1993). V1 layer 2/3 projects up to V2 layers 6 and 4, just as LGN projects to layers 6 and 4 of V1. Higher cortical areas send feedback into V2 which ultimately reaches layer 6, just as V2 feedback acts on layer 6 of V1 (Sandell and Schiller, 1982). Feedback paths from higher cortical areas straight into V1 (not shown) can complement and enhance feedback from V2 into V1 (reprinted with permission from Raizada and Grossberg, 2001).

The modeling of how ART mechanisms may be embodied within known laminar microcircuits of the cerebral cortex began in Grossberg (1999). This laminar version of ART is called LAMINART (Figure 5). The LAMINART embedding is not a mere relabeling of the previous ART theory. Rather, this unlumping of the previous non-laminar ART model resolved a long-standing conceptual problem and enabled the explanation and prediction of much more psychological and brain data. See Grossberg (2013; sections 26 and 27) for a review. In so doing, it unified two major streams of research activity:

(1) ART as a theory of category learning and prediction. As reviewed above, this stream emphasized bottom-up and top-down interactions during the learning of visual recognition categories by higher-level cortical circuits such as cortical areas V4, inferotemporal cortex and PFC;
(2) FACADE (Form-And-Color-And-DEpth) as a theory of 3D vision and figure-ground perception (e.g., Grossberg, 1994, 1997; Grossberg and McLoughlin, 1997; Grossberg et al., 2007). This stream emphasized bottom-up and horizontal interactions for completion of boundaries during perceptual grouping, and for filling-in of surface brightness and color by lower cortical processing areas such as V1, V2 and V4.

The unification of these two research streams in LAMINART proposed how *all* neocortical areas combine bottom-up, horizontal, and top-down interactions, thereby clarifying in all granular neocortical areas functional roles for the identified cells in the six main cell layers of such cortices (Felleman and Van Essen, 1991). It was also shown how this shared laminar circuit design may be specialized to carry out qualitatively different kinds of biological intelligence, and used them to explain and predict psychological and neurobiological data about vision, speech and cognition:

- ***Vision***. 3D LAMINART integrates bottom-up and horizontal processes of 3D boundary formation and perceptual grouping, surface filling-in, and figure-ground separation with top-down attentional matching in cortical areas such as V1, V2 and V4 (e.g., Grossberg and Howe, 2003; Grossberg and Swaminathan, 2004; Cao and Grossberg, 2005, 2012; Grossberg and Yazdanbakhsh, 2005; Grossberg et al., 2008b; Fang and Grossberg, 2009; Léveillé et al., 2010).
- ***Speech***. cARTWORD models how bottom-up, horizontal and top-down interactions within a hierarchy of laminar cortical processing stages, modulated by the basal ganglia, can generate a conscious speech percept that is embodied by a resonant wave of activation that occurs between acoustic features, item chunks and list chunks (Grossberg and Kazerounian, 2011; Kazerounian and Grossberg, 2014). Chunk-mediated gating via the basal ganglia allows speech to be heard in the correct temporal order, even when what is consciously heard depends upon using future context to disambiguate noise-occluded sounds, as occurs during phonemic restoration.
- ***Cognition***. LIST PARSE models how bottom-up, horizontal, and top-down interactions within the laminar circuits of lateral PFC may carry out working memory storage of event sequences within layers 6 and 4, unitization of these event sequences through learning of list, or sequence, categories within layer 2/3, and recall of the stored sequences at variable rates that are under volitional control by the basal ganglia (Grossberg and Pearson, 2008). In particular, the model uses variations of the same circuitry to quantitatively simulate human cognitive data about immediate serial recall and immediate, delayed, and continuous distractor free recall; and monkey neurophysiological data from the PFC obtained during sequential sensory-motor imitation and planned performance.

## 8 Smart: ACh Vigilance Control in Laminar Cortical Art Models with Spiking Neurons

### 8.1 From Rate-Based to Spiking ART Models

**Figure 6:**
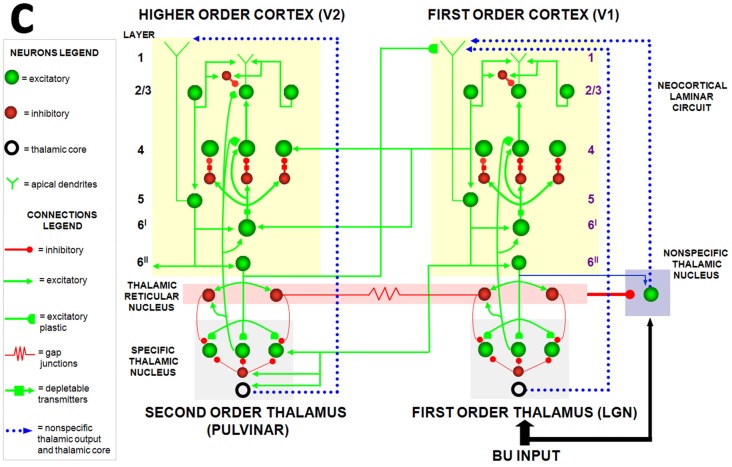
The Synchronous Matching ART, or SMART, model clarifies how laminar neocortical circuits in multiple cortical areas interact with specific and nonspecific thalamic nuclei to regulate learning on multiple organizational levels, ranging from spikes to cognitive dynamics. The thalamus is subdivided into specific first-order and second-order nuclei, nonspecific nucleus, and thalamic reticular nucleus (TRN). The first-order thalamic matrix cells (shown as an open ring) provide nonspecific excitatory priming to layer 1 in response to bottom-up input, priming layer 5 cells and allowing them to respond to layer 2/3 input. This allows layer 5 to close the intracortical loop and activate the pulvinar (PULV). V1 layer 4 receives inputs from two parallel bottom-up thalamocortical pathways: a direct LGN→4 excitatory input, and a 6^I^→4 modulatory on-center, off-surround network that contrast-normalizes the pattern of layer 4 activation via the recurrent 4→2/3→5→6^I^→4 loop. V1 activates the bottom-up V1→V2 corticocortical pathways from V1 layer 2/3 to V2 layers 6^I^ and 4, as well as the bottom-up corticothalamocortical pathway from V1 layer 5 to the PULV, which projects to V2 layers 6^I^ and 4. In V2, as in V1, the layer 6^I^→4 pathway provides divisive contrast normalization to V2 layer 4 cells. Corticocortical feedback from V2 layer 6^II^ reaches V1 layer 1, where it activates apical dendrites of layer 5 cells. Layer 5 cells, in turn, activate the modulatory 6^I^→4 pathway in V1, which projects a V1 top-down expectation to the LGN. TRN cells of the two thalamic sectors are linked via gap junctions, which synchronize activation across the two thalamocortical sectors when processing bottom-up stimuli. The nonspecific thalamic nucleus receives convergent bottom-up excitatory input from specific thalamic nuclei and inhibition from the TRN, and projects to layer 1 of the laminar cortical circuit, where it regulates mismatch-activated reset and hypothesis testing in the cortical circuit. Corticocortical feedback connections from layer 6^II^ of the higher cortical area terminate in layer 1 of the lower cortical area, whereas corticothalamic feedback from layer 6^II^ terminates in its specific thalamus and on the TRN. This corticothalamic feedback is matched against bottom-up input in the specific thalamus (reprinted with permission from Grossberg and Versace, 2008).

The LAMINART model was developed in articles such as Grossberg and Raizada (2000) and Raizada and Grossberg (2001) to simulate psychological and neurobiological data about 2D visual perception using rate-based neurons. 3D LAMINART unlumped, and generalized, these analyses to the perception of objects in depth. LAMINART was also unlumped in another direction by the Synchronous Matching ART, or SMART, model (Figure 6; Grossberg and Versace, 2008; Grossberg et al., 2016) wherein rate-based neurons were replaced with spiking neurons, among other refinements.

**Figure 7:**
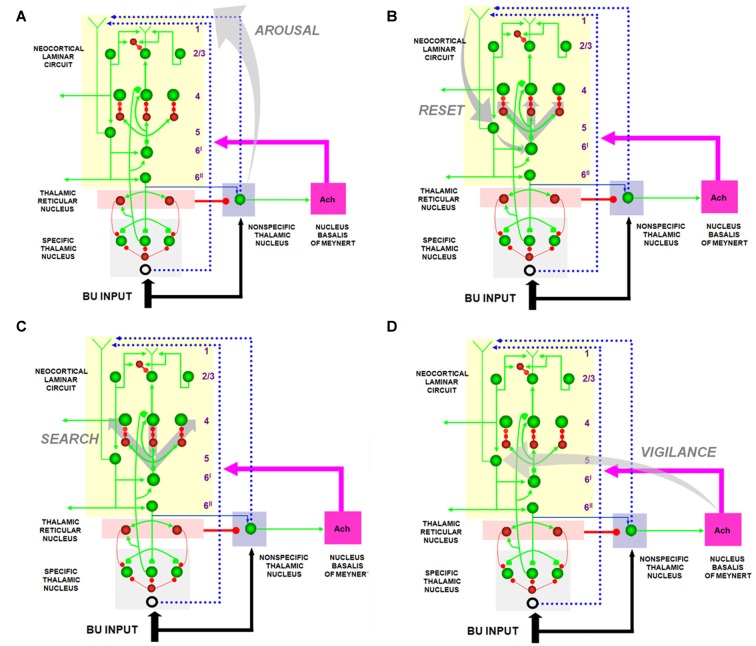
The large shaded gray arrows in the figure indicate the SMART pathways involved in the generation of the **(A)** *AROUSAL* burst, **(B)** *RESET*, **(C)** *SEARCH* and **(D)**, *VIGILANCE* control. See text for details (reprinted with permission from Grossberg and Versace, 2008).

The SMART model predicted that vigilance is controlled by modifying the excitability of cortical layer 5 cells using acetylcholine (ACh) that is released there in response to mismatch events (Figure 7). The anatomical pathway leading to ACh release has been clarified by anatomical and neurophysiological studies in monkeys, cats and rats. These studies show that the nonspecific thalamus—in particular, the midline and central lateral thalamic nuclei—are sensitive to the degree of mismatch (Figures 6, 7), and project to the cholinergic nucleus basalis of Meynert (Figure 7; Van der Werf et al., 2002) which, in turn, is one of the main sources of cholinergic innervation to layer 5 of the cerebral cortex.

How ACh may influence vigilance is illustrated by experiments of Saar et al. (2001), who have shown that ACh release reduces the after-hyperpolarization (AHP) current and increases cell excitability in cortical layer 5 cortical cells. In SMART, this increased layer 5 excitability due to predictive mismatch may cause reset via the layer 5-to-6^I^-to-4 circuit (Figures 6, 7), even in cases where top-down feedback may earlier have sufficiently matched bottom-up input, which is a key property of vigilance control. An increase of ACh might hereby trigger a search for finer recognition categories in response to environmental feedback, even when bottom-up and top-down signals have a pretty good match in the nonspecific thalamus based on similarity alone.

### 8.2 Match-Induced Gamma Oscillations and Mismatch-Induced Beta Oscillations

Grossberg and Versace (2008) used the SMART model to make other predictions that are related to the ACh-modulated vigilance prediction, and that may be combined with it to design new kinds of experimental tests. In particular, within SMART, a good enough top-down attentive match with a bottom-up feature pattern yields (faster) gamma oscillations, whereas a big enough mismatch-and-reset yields (slower) beta oscillations. The model also predicts how the mismatch is triggered in deeper layers of cortex (Figure 7B) before the reset can reorganize processing in all the cortical layers (Figure 7C). Both types of oscillations are emergent properties of network dynamics.

Grossberg (2013, Section 38) reviews the fact that this predicted match–mismatch gamma–beta dichotomy has subsequently been supported by neurophysiological experiments from at least three laboratories recording in three different parts of the brain. These reports include data about higher amounts of average gamma in superficial cortical layers vs. more beta (or alpha) power in deep cortical layers of cortical area V1 (Buffalo et al., 2011), beta synchronized with spatial attention shifts in the frontal eye fields (Buschman and Miller, 2009), and an inverted-U in beta power through time during learning of place cells in hippocampus during the navigation of novel spatial environments (Berke et al., 2008). Combining the gamma-beta prediction with the ACh-vigilance prediction suggests that an ACh-modulated increase of vigilance will, other things being equal, reduce the amount of gamma relative to beta power by increasing the number of mismatch reset events per unit time.

The match–mismatch gamma–beta prediction can also be tested by redoing the experiments of Spitzer et al. (1988) in which neurophysiological data are recorded from cortical area V4 during the learning by monkeys of easy vs. difficult discriminations. These authors report that “in the difficult condition, the animals adopted a stricter internal criterion for discriminating matching from non-matching stimuli… The animal’s internal representations of the stimuli were better separated… increased effort appeared to cause enhancement of the responses and sharpened selectivity for attended stimuli…” These are all properties of ART vigilance control, where higher vigilance is needed to make more difficult discriminations. These V4 effects could, for example, be due to vigilance-modulated selective attention from inferotemporal cortex (IT). Such object attention is focused via vigilance-modulated top-down expectations that are learned along with the bottom-up adaptive pathways that select IT recognition categories. Other things being equal, one would expect to find more beta power in the difficult than the easy discrimination condition. Direct activation of nucleus basalis or appropriate ACh target neurons might also be expected to increase beta power.

### 8.3 How ACh Modulates AHP Currents in Spiking Networks to Regulate Vigilance

How does a reduction of AHP current by ACh increase cell excitability in cortical layer 5 cortical cells (Saar et al., 2001)? Many cortical networks contain recurrent on-center off-surround networks that transform input patterns before storing them in STM. A series of theorems in the 1970s (e.g., Grossberg, 1973; Grossberg and Levine, 1975) proved how the feedback signal functions in rate-based recurrent on-center off-surround networks control this STM storage process. In particular, sigmoid signal functions in such networks induce a *quenching threshold* below which inputs are suppressed as noise and above which they are contrast-enhanced before being stored in STM. A sudden decrease of the quenching threshold, other things being equal, can cause a reset burst and thus act like an increase in vigilance.

What action of ACh modulation could cause a sudden decrease in quenching threshold? If a mismatch-mediated burst of ACh suitably changes the shape of a sigmoid signal function, say by decreasing its threshold and increasing its slope, then that may decrease the quenching threshold.

A sigmoid signal can be explicitly defined in a rate-based neural network. In contrast, in an unlumped network composed of spiking neurons, the shape of the signal function is an emergent property of several factors that interact together. Palma et al. (2012a,b) showed how AHP currents that act on three different time scales can together control sigmoid signal threshold and slope in networks of spiking neurons. These properties clarify how activation of ACh by basal forebrain circuits, notably nucleus basalis of Meynert, may alter the brain’s sensitivity to predictive mismatches, and thus the vigilance with which the learning of recognition categories is modulated in the brain.

These AHP currents are predominantly carried by calcium-dependent potassium channels (Hotson and Prince, 1980; Lancaster and Adams, 1986), but also partly by calcium-independent potassium currents (Lorenzon and Foehring, 1992, 1995). In layer 5 Betz cells of cat sensorimotor cortex (Schwindt et al., 1988) and pyramidal cells in layers 3–6 of human neocortex (Lorenzon and Foehring, 1992) three distinct AHP currents—a fast (fAHP), medium (mAHP), and slow current (sAHP)—have been identified. Similar mAHP and sAHP currents occur in rat slices (Storm, 1987, 1989; Lee et al., 2005). How the different time courses of these AHP currents arise has not been completely explained, but proximity to calcium channels may be a key factor rather than the time constant of calcium binding to the channels themselves (Lima and Marrion, 2007).

The Palma et al. (2012b) simulations demonstrated that, in spiking neurons, a leftward threshold shift in the sigmoid signal occurs when the sAHP and mAHP currents decrease, as the fAHP current increases, whereas the slope of the transfer function becomes steeper when the sAHP and fAHP currents decrease, as the mAHP current increases. Both of these changes can reduce the quenching threshold and thereby increase vigilance.

## 9 Sustained vs. Transient Vigilance Control by ACh

Vigilance can change over multiple timescales, from the rapid transients during pattern matching processes to the contextual or task-based setting of baseline vigilance levels. That ACh concentration transients can occur rapidly at the timescale of a behavioral episode has been demonstrated in several articles (e.g., Sarter et al., 2005; Parikh and Sarter, 2006; Parikh et al., 2007). On slower timescales, ACh levels are known to oscillate with circadian rhythms (Williams et al., 1994; Marrosu et al., 1995; Crouzier et al., 2006), increase with caffeine administration through its action as an adenosine receptor antagonist (Carter et al., 1995; Kurokawa et al., 1996), and vary in a task-dependent manner that correlates with attentional demands as confirmed by microdialysis (Marrosu et al., 1995; Arnold et al., 2002) and newer techniques (Parikh et al., 2007). The variation of ACh levels with circadian rhythms are relevant to the discussion in Section 11 of a link between ACh and sleep.

These results are consistent with reports that basal forebrain neurons, notably their cholinergic projections, are involved in both tonic and phasic activations of the cerebral cortex, including a close link between basal forebrain activity and the EEG (Détári et al., 1999).

The largest fluctuations in vigilance may be expected during tasks that require rapid learning of novel information in an ART system. In this regard, it is known that activity of the nucleus basalis of Meynert facilitates plasticity of cortical maps both in primary auditory cortex (Kilgard and Merzenich, 1998) and in motor cortex (Ramanathan et al., 2009). ART predicts that the highest levels of vigilance, and consequently ACh, should occur when incorporating novel exemplars into memory during mismatch processing, while lower levels of ACh may be sufficient to refine category representations during match episodes. Correspondingly, studies of how scopolamine influences human memory formation suggest that high levels of ACh promote rapid encoding, whereas low levels of ACh support consolidation (Rasch et al., 2006). Lowering ACh with scopolamine has also been proposed to improve memory consolidation by preventing possible interference with conflicting information (Winters et al., 2007). Interference in both cases could be interpreted as learning of categories that are too general for the more difficult task.

## 10 ACh Modulation of Learned Category Generality Through Time

The baseline vigilance in ART sets the criterion for expectation mismatch and novelty detection, and thereby indirectly determines learned category generality, with low baseline vigilance favoring general, or abstract, categories and high baseline vigilance more specific, or concrete, categories. General categories may fail to discriminate when a task involves pattern interference that may be due to featural overlap across categories. Correspondingly, object discrimination studies in rats show that scopolamine reduces the novelty discrimination ratio (Ballaz, 2009).

In this regard, other experiments also suggest that fluctuations in cortical ACh are necessary for learning mainly when there is featural overlap during perceptual categorization (Chiba et al., 1995; Atri et al., 2004; Botly and De Rosa, 2007, 2009; Hata et al., 2007; Winters et al., 2007), consistent with the ART prediction that ACh can increase vigilance to achieve better categorical separation.

For example, lesions in rats of the nucleus basalis of Meynert have little impact on learning rate, except when high interference can occur between the categories to be learned because they share the same features in a certain dimension (Botly and De Rosa, 2007, 2009). Studies in humans also show that scopolamine, by competitively binding muscarinic receptors, diminishes learning of overlapping word pairs more than non-overlapping pairs (Atri et al., 2004). Associative learning studies in rats with combinations of light and tone report that the concentration of released ACh increases more during discrimination learning experiments in which an individual stimulus (A, e.g., light) signals reward and a compound stimulus (AB, e.g., light + tone) signals no reward, than during element discrimination, in which one stimulus (A, e.g., light) signals reward and another stimulus (B, e.g., tone) signals no reward (Hata et al., 2007). Finally, donepezil, which increases cortical ACh by inhibiting its degradation by Acetylcholinesterase (AChE), has been shown by fMRI to reduce the expanse of response in V1 from a pulsating visual stimulus (Silver et al., 2008). Taken together, these data suggest that increased ACh, and with it better focused attention, help to cause more selective categorical representations to form.

ACh release may also cause desynchronization between cortical cells (Goard and Dan, 2009) in conjunction with up-regulation of high gamma oscillations (Canolty et al., 2006). Other experiments suggest that both this desynchronization (Pandya et al., 2005) and gamma oscillation regulation (Keizer et al., 2010) may be crucial for interference learning. Further experiments would be needed to determine if the desynchronization corresponds to predicted ART mismatch-reset and beta oscillations in response to increased ACh-modulated vigilance, followed by gamma oscillations during the next match state.

Further experiments would also be needed to investigate the following issue: How is the baseline vigilance determined in environments that contain few novel events and/or easy discriminations vs. in environments that contain many novel events and/or difficult discriminations? One might imagine, if baseline vigilance is at all sensitive to such environmental statistical factors, that it increases in environments that include many novel events and/or difficult discriminations. Such a change in baseline vigilance could be achieved by a process of automatic gain control. Here, each mismatch event would add to a time-average of mismatch bursts (the “gain”) that increments the baseline vigilance when novelty and/or difficulty are high, and decrements the baseline vigilance when novelty and/or difficulty are low. Similar automatic gain control processes seem to occur in many brain processes that respond to changing environmental statistics. For example, automatic gain control can speed up or slow down the speech processing rate in response to the average rate of speech in different environments, and thereby supports the learning of a rate-invariant code for language meaning (Repp, 1980; Grossberg et al., 1997; Grossberg, 2017).

## 11 ACh, Vigilance, Nucleus Basalis and Slow Wave Sleep

### 11.1 Some Sleep Data

At the other end of the continuum from high vigilance and environmentally-sensitive attention, learning, and recognition are the complex phenomena that occur during sleep. A full analysis of the complexities of sleep is not attempted here. Rather, relevant sleep data are summarized to set the stage for the proposal that specific properties of laminar neocortical circuits that have been used to simulate data about perception and cognition in awake individuals (Figures 5–7) may also shed light on the dynamics of slow wave sleep. This proposed connection suggests that these properties of sleep may emerge from vigilance changes in the same model laminar neocortical circuits that have successfully modeled various awake behaviors.

Relevant sleep data include the following: Steriade (2004, p. 179) has noted that: “Fast rhythms (20–60 Hz) appear during the sustained depolarization of thalamic and neocortical neurons during brain-active states that are accompanied by increased release of ACh in the thalamus and cerebral cortex. Such fast rhythms also occur during the depolarizing phases of the slow oscillation (0.5–1 Hz) in non-REM sleep. Intracellular recordings of neocortical neurons during natural states of waking and sleep demonstrate stable and increased input resistance of corticocortical and corticothalamic neurons during the sustained depolarization in wakefulness, compared to the depolarizing phase of the slow oscillation in non-REM sleep. Despite the highly increased synaptic inputs along different afferent systems that open many conductances of cortical neurons during wakefulness, the increased input resistance is attributed to the effect of ACh on cortical neurons”.

An earlier study of Metherate et al. (1992, p. 4701) reported compatible data: “In the mammalian neocortex, the EEG reflects the state of behavioral arousal. The EEG undergoes a transformation, known as activation, during the transition from sleep to waking. Abundant evidence indicates the involvement of the neurotransmitter acetylcholine (ACh) in EEG activation; however, the cellular basis of this involvement remains unclear. We have used electrophysiological techniques with *in vivo* and *in vitro* preparations to demonstrate actions of endogenous ACh on neurons in auditory neocortex. *In vivo* stimulation of the nucleus basalis (NB), a primary source of neocortical ACh: (1) elicited EEG activation via cortical muscarinic receptors; (2) depolarized cortical neurons; and (3) produced a change in subthreshold membrane potential fluctuations from large-amplitude, slow (1–5 Hz) oscillations to low-amplitude, fast (20–40 Hz) oscillations. The NB-mediated change in pattern of membrane potential fluctuations resulted in a shift of spike discharge pattern from phasic to tonic. Stimulation of afferents in the *in vitro* neocortex elicited cholinergic actions on putative layer 5 pyramidal neurons. Acting via muscarinic receptors, endogenous ACh: (1) reduced slow, rhythmic burst discharge and facilitated higher-frequency, single-spike discharge in burst-generating neurons; and (2) facilitated the appearance and magnitude of intrinsic membrane potential oscillations. These *in vivo* and *in vitro* observations suggest that neocortical activation results from muscarinic modulation of intrinsic neural oscillations and firing modes…”

Further quantification of the role of basal forebrain ACh release during wakefulness and sleep was provided by the study of Vazquez and Baghdoyan (2001, p. R598) who reported that: “Cortical ACh release is greatest during waking and rapid eye movement (REM) sleep and reduced during non-REM (NREM) sleep. Loss of basal forebrain cholinergic neurons contributes to sleep disruption and to the cognitive deficits of many neurological disorders. ACh release within the basal forebrain previously has not been quantified during sleep. This study used *in vivo* microdialysis to test the hypothesis that basal forebrain ACh release varies as a function of sleep and waking. Cats were trained to sleep in a head-stable position, and dialysis samples were collected during polygraphically defined states of waking, NREM sleep, and REM sleep. Results from 22 experiments in four animals demonstrated that means ± SE ACh release (pmol/10 min) was greatest during REM sleep (0.77 ± 0.07), intermediate during waking (0.58 ± 0.03), and lowest during NREM sleep (0.34 ± 0.01)…”. The fact that ACh release was greater during REM sleep than wakefulness is a reminder that conscious awareness does not depend only on ACh modulation. Neural models describe some of the other brain processes that are proposed to play a key role during conscious awareness. For recent reviews, see Grossberg (2016, 2017).

More recent experiments provide further details confirming the causal relationship between cholinergic neurons of the basal forebrain and sleep homeostasis (e.g., Kalinchuk et al., 2015; Nair et al., 2016).

### 11.2 Laminar Cortical Model Circuits Unify Properties of Sleep and Awake Dynamics

This section and the next one describe how laminar cortical ART models that provide a unified explanation in the awake individual of processes of boundary completion, contrast normalization, attentional modulation, decision-making, activity-dependent habituation, and mismatch-mediated reset, also exhibit properties of Up and Down states during slow wave sleep. First some additional data about slow wave sleep are reviewed before suggesting how they may arise from mechanisms that have earlier been characterized for the above processes in awake individuals.

As noted above, NREM sleep often exhibits a slow <1 Hz rhythm in the EEG that appears to have a cortical origin (Steriade, 1991; Steriade et al., 1993a,b; Timofeev and Steriade, 1996; Sanchez-Vives and McCormick, 2000; Crunelli and Hughes, 2010; Sanchez-Vives and Mattia, 2014). This slow rhythm has been proposed to carry out multiple functions, ranging from metabolic clearance from the brain (Xie et al., 2013) to memory consolidation (Steriade and Timofeev, 2003; Marshall et al., 2006; Franklin and Grossberg, 2017). Lesions of the basal forebrain cholinergic nuclei, or pharmacological block of muscarinic receptors, lead to such slow waves throughout the neocortex (Buzsaki et al., 1988; Vanderwolf, 1988).

Slow wave generation in layer 5 (Calvet et al., 1973; Ball et al., 1977; Rappelsberger et al., 1982) is supported by bursting pyramidal cells in layer 5 that synchronize activity across the neocortex (e.g., Connors, 1984; Chagnac-Amitai and Connors, 1989; Silva et al., 1991; Wang and McCormick, 1993). Intrinsically bursting cells have also been reported in layer 3 (Steriade et al., 1993a,b). In the transition from sleep to wakefulness, this spontaneous burst firing is replaced by the spike sequences that are frequency-modulated by input intensities and related parameters. Livingstone and Hubel (1981) have, for example, reported a marked increase in activity in layers 5 and 6 during wakefulness as compared to during sleep. This transition is supported by a significant increase in the extracellular concentration of ACh (Szerb, 1967; Phillis, 1968).

An important property of the Up and Down states of slow wave sleep is that, as Volgushev et al. (2006, p. 5671) have observed: “all cells, excitatory as well as inhibitory, were involved in the same slow rhythm, and we never observed a cell to be systematically active while other neurons were silent…high synchrony of the silent state onsets implies the existence of a network mechanism that switches activity to silence… This would lead to termination of activity of both excitatory and inhibitory cells, including those cells that have generated the silencing discharge”. These authors go on to review evidence that bursting neurons in layer 5 large pyramidal cells initiate this activity cycle, and that fast-spiking inhibitory interneurons have an early onset during the subsequent Up and Down states. These dynamics are, moreover, due to intracortical interactions that occur even when thalamic gates do not permit the intrusion of signals from the outside world during slow wave sleep (e.g., Steriade and Timofeev, 2003). An intracortical origin for these slow waves is also supported by their survival after extensive thalamic lesions (Steriade et al., 1993b) and by the absence of slow waves in the thalamus of decorticated cats (Timofeev and Steriade, 1996). In their mean field model of the Up-Down cycle, Sanchez-Vives and Mattia (2014) have also observed a role for “activity-dependent fatigue” in regulating the relative duration of the Down state.

In order to more fully understand how such properties of slow wave sleep may arise, it is informative to link them to laminar neocortical circuits that also play important functional roles during the awake state. Ideally, many properties of slow wave sleep could then be shown to arise as natural emergent properties of these cortical circuits, in just the same way that their properties during awake states do. Such a connection will be outlined below. This proposed link between awake and sleep states also sheds some light on the following questions: Why do not the bursts that occur during slow wave sleep cause movement, let alone consciousness, especially given that layer 5 cells project to movement causing areas of the brain such as the superior colliculus and spinal cord, as well as subcortical targets such as the pontine nuclei (Wang and McCormick, 1993)? Multiple mechanisms may contribute to this result, but it is shown here how a prescribed intracortical mechanism may also play a role.

Several experimental and modeling studies have contributed insights into the cellular mechanisms of slow wave sleep and how it may transition to the waking state (e.g., Bazhenov et al., 2002; Esser et al., 2009). These studies have not, however, tried to connect their studies of sleep to laminar cortical models that have been used to quantitatively simulate psychological and neurophysiological data that have been collected during awake behaviors. Such a connection is made here, and adds new concepts and circuit mechanisms to this discussion. In particular, these are *network* mechanisms that seem to be conserved in multiple neocortical regions, and that can help to explain synchronization and wave-like properties of slow wave sleep across the neocortex (Massimini et al., 2004; Volgushev et al., 2006).

### 11.3 Self-Normalizing Laminar Cortical Circuits Balance Excitation and Inhibition

The discussion in this section will focus on the laminar cortical circuits in the visual cortex that are modeled by the LAMINART model, and its 3D LAMINART extension. Similar laminar circuits, with suitable specializations, have been used to simulate speech perception data using the cARTWORD model, and data about cognitive working memory and list chunking using the LIST PARSE model (Section 7). These mechanisms thus seem to be conserved, albeit with suitable specializations to carry out different psychological functions, across at least the granular neocortex, with its characteristic architecture of six layers of cells and their sublaminae, and the characteristic bottom-up, top-down, and horizontal interactions that enable their functional capabilities (Brodmann, 1909; Martin, 1989).

Two kinds of interactions within layers 2/3 and 5/6 are of special interest to the explanatory goals herein: Long-range excitatory horizontal connections, and shorter-range self-normalizing networks that balance excitatory and inhibitory interactions. The long-range horizontal connections within cortical layers 2/3 in LAMINART carry out the process of boundary completion, also called perceptual grouping (e.g., Grossberg and Raizada, 2000; Raizada and Grossberg, 2001, 2003; Grossberg and Swaminathan, 2004; Grossberg and Yazdanbakhsh, 2005; Fang and Grossberg, 2009; Léveillé et al., 2010). In addition, perceptual grouping can support a traveling wave in response to a spatial focus of attention (Grossberg and Raizada, 2000, Figure 3; Roelfsema et al., 1998). Shorter-range excitatory horizontal connections in layers 2/3 and 5/6 carry out processes such as binocular fusion (Grossberg and Howe, 2003, Figure 18). These features of cells in cortical layers 2/3 and 5/6 take on new significance in the context of sleep research for at least two reasons: (1) layers 3 and 5 contain intrinsically bursting neurons that may be influenced by ACh modulation (Calvet et al., 1973; Ball et al., 1977; Rappelsberger et al., 1982; Connors, 1984; Chagnac-Amitai and Connors, 1989; Silva et al., 1991; Steriade et al., 1993a,b; Wang and McCormick, 1993); (2) long-range excitatory connections may help to both synchronize the ACh-modulated slow waves within local cortical networks, and to facilitate the propagation of these synchronous activations in traveling waves across the cortex (Massimini et al., 2004; Volgushev et al., 2006).

**Figure 8:**
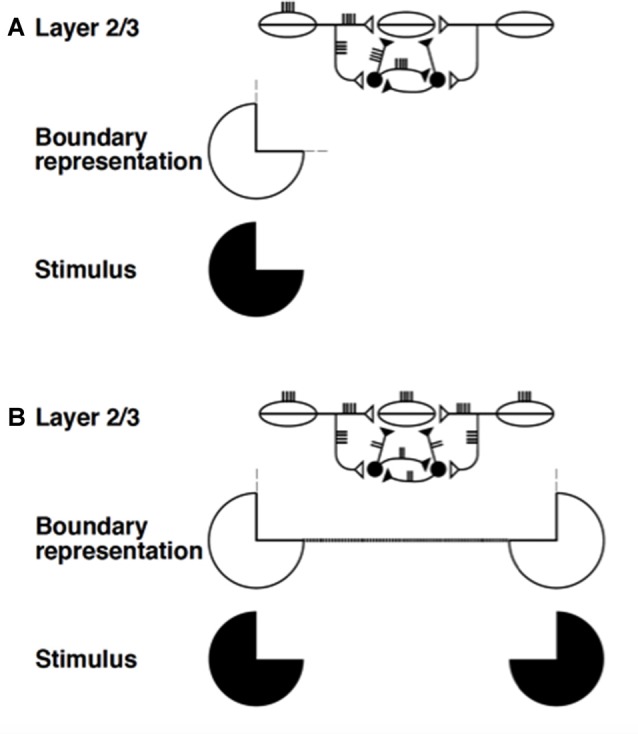
Summary of boundary completion dynamics in layer 2/3. Pyramidal cells with collinear, coaxial receptive fields (shown as ovals) excite each other via long-range horizontal axons (Bosking et al., 1997; Schmidt et al., 1997), which also give rise to short-range, disynaptic inhibition via pools of interneurons, shown filled-in black (McGuire et al., 1991). This balance of excitation and inhibition helps to implement the bipole property: **(A)** Horizontal input coming in from just one side is insufficient to cause above-threshold excitation in a pyramidal cell (henceforth referred to as the target) whose receptive field does not itself receive any bottom-up input. The inducing stimulus (e.g., a Kanizsa pac man, shown here) excites the oriented receptive fields of layer 2/3 cells, which send out long-range horizontal excitation onto the target pyramidal cell. However, this excitation brings with it a balanced amount of disynaptic inhibition. This creates a case of “one-against-one”, and the target pyramidal is not excited above threshold. The boundary representation of the solitary pac man inducer produces only weak, subthreshold collinear extensions (thin dashed lines). **(B)** When two collinearly aligned inducing stimuli are present, one on each side of the target pyramidal cell’s receptive field, a boundary grouping can form. Long-range excitatory inputs from both sides summate. However, these inputs activate a shared pool of inhibitory interneurons, which, as well as inhibiting the target pyramidal, also inhibit each other (Tamás et al., 1998), thus normalizing the total amount of inhibition emanating from the interneuron pool, without any individual interneuron saturating. Summating excitation and normalizing inhibition together create a case of “two-against-one”, and the target pyramidal is excited above threshold. This process occurs along the whole boundary grouping, which thereby becomes represented by a line of suprathreshold layer 2/3 cells (thick dotted line). Boundary strength scales in a graded analog manner with the strength of the inducing signals (reprinted with permission from Raizada and Grossberg, 2001).

The networks with self-normalizing balanced excitatory and recurrent inhibitory interneurons are at least as important from the present perspective. They occur, with a similar design, in both layers 2/3 and 5/6. In layers 2/3, they are part of a larger circuit that also includes the long-range excitatory horizontal connections. Figures 5C,E simplify the depiction of the inhibition in layer 2/3 as a single inhibitory interneuron. Together with the long-range horizontal connections, the shorter-range recurrent inhibitory network helps to realize the *bipole property* that enables perceptual grouping to occur in the following way (Figure 8; Grossberg, 1984a; von der Heydt et al., 1984; Grossberg and Mingolla, 1985; Peterhans and von der Heydt, 1989).

In Figure 8A, a single pac man figure (in black) induces a boundary representation using simple and complex cells in cortical area V1 whose orientation preferences are the same as, or similar to, those of the pac man’s bounding contour. This boundary representation activates long-range excitatory horizontal connections in layer 2/3 of cortical area V2. Excitatory signals in these horizontal connections also excite inhibitory interneurons on their way to target cells in layer 2/3. The inhibition from the disynaptic inhibitory interneurons balances the excitation from the long-range horizontal connection, thereby preventing the target cell from firing. It is a case of one-against-one. By this mechanism, a single contrast in an image is prevented from creating outwardly spreading boundaries across the entire image.

In Figure 8B, two like-oriented pac man figures are aligned across space, and are sufficiently near one another to complete a boundary between them. In this case, the excitatory signals from their long-range horizontal connections summate at the target cell. The disynaptic inhibitory signals from the inhibitory interneurons also summate there. Why, then, does not the total inhibition again cancel the total excitation? This is because—and this is the main point—the inhibitory interneurons also inhibit each other to form a recurrent lateral inhibitory network. It was mathematically proved in Grossberg (1973; see also Grossberg, 1980) that, if the inhibitory cells in such a recurrent network obey the membrane equations of neurophysiology—also called shunting dynamics—then their *total activity tends to be normalized*, and independent of the number of active inhibitory cells. Thus, although the total excitation increases, the total inhibition does not. It is a case of two-against-one, so excitation wins and the boundary can form.

Layers 5/6 also include the same kind of recurrent self-normalizing circuit. This kind of circuit, in fact, seems to occur in many parts of the neocortex. In Figures 5A–C,E, these inhibitory interneurons are drawn as a feedforward network, for simplicity. In actuality, they also inhibit each other in the LAMINART model, thereby creating self-normalizing total inhibition. This property helps to ensure properties in the adult of contrast normalization, attentional modulation, decision-making, activity-dependent habituation, and mismatch-mediated reset in a wide variety of visual processes (e.g., Francis et al., 1994; Francis and Grossberg, 1996; Grossberg, 2003, 2016; Cao and Grossberg, 2005, 2012; Grossberg and Yazdanbakhsh, 2005; Grossberg and Pilly, 2008; Grossberg and Versace, 2008).

### 11.4 Why Are Self-Normalizing Circuits Needed?

The need for such self-normalizing inhibition can be understood by considering Figure 5A. Here, the network from layer 6-to-4 is supposed to create a *modulatory* on-center as part of the on-center off-surround network that inputs to layer 4. Such a modulatory on-center requires that excitatory and inhibitory inputs are approximately equal, or balanced, to the on-center cells. The lateral geniculate nucleus (LGN) is able to fire target cells in layer 4 by also activating a direct LGN-to-4 excitatory pathway. The indirect LGN-to-6-to-4 is not useless, however. The direct LGN-to-4 pathway, combined with the LGN-to-6-to-4 network, can both activate and *contrast-normalize* the inputs to layer 4 cells in response to input patterns from the LGN.

Why is the combination of a direct LGN-to-4 network and a modulatory on-center off-surround network from LGN-to-6-to-4 needed? Why could not the LGN-to-6-to-4 network be designed so that its excitatory on-center could, all by itself, fire target layer 4 cells, thereby eliminating the need for a separate LGN-to-4 pathway? This can be understood by considering how the modulatory on-center in the 6-to-4 network helps to realize top-down attention via the ART Matching Rule in Figure 5B. As noted in Section 2.2, the ART Matching Rule asserts that top-down object attention is achieved using a top-down, *modulatory* on-center, off-surround network *in order to achieve stable learning and memory*. The excitation and inhibition in the on-center of the layer 6-to-4 network thus need to be approximately balanced. This constraint clarifies the need for a direct LGN-to-4 pathway that is sufficient to fire target layer 4 cells.

### 11.5 Recurrent Off-Surround Normalizes Cell Responses to Converging Sources

The above considerations explain why the layer 6-to-4 on-center is modulatory, but not why the off-surround needs to be a *recurrent* inhibitory network. The main design constraint that forces recurrence is that the modulatory requirement must be maintained both when multiple input sources contribute simultaneously to the total top-down attentional priming signal, or to the total bottom-up feature pattern. Under these variable load conditions, the modulatory requirement would fail if the inhibitory network was feedforward. In particular, if the total activity of off-surround cells grew proportionally with the number of cells inputting to it, then the excitatory inputs to the on-center would be overwhelmed. Self-normalization of total inhibition by the recurrent inhibitory network, no matter how many input sources are active, solves this problem.

Balancing excitation and inhibition in these circuits achieves several other useful functional roles. For starters, it helps to avoid seizures that could result if excitation was too much stronger than inhibition, or persistent depression if inhibition was too much stronger than excitation. Such a balance also maintains the cortex at a “cusp of excitability” wherein enough activation occurs to maintain homeostatic plasticity without triggering undesired behavioral consequences, while also enabling the cortex to respond efficiently and vigorously to external inputs when they do occur. Indeed, the laminar cortical model of Grossberg and Williamson (2001) showed how the desired balance of excitation and inhibition could develop for carrying out bipole grouping circuits in layers 2/3 and contrast-normalizing attentional modulation in layers 6-to-4. These developed circuits were then used in that article and subsequent ones (e.g., Grossberg and Raizada, 2000; Raizada and Grossberg, 2001) to simulate a wide range of psychophysical and neurophysiological data about adult visual perception and attention, thereby showing how LAMINART models can provide a unified explanation of data about both cortical development and adult attention and visual perception.

It is also worth noting that a recurrent inhibitory network is probably easier to grow during development than a feedforward network, since inhibitory connections can then grow to *all* nearby cells, both excitatory and inhibitory, not just to excitatory cells.

### 11.6 From Grouping and Attention to Up and Down States during Slow Wave Sleep

**Figure 9:**
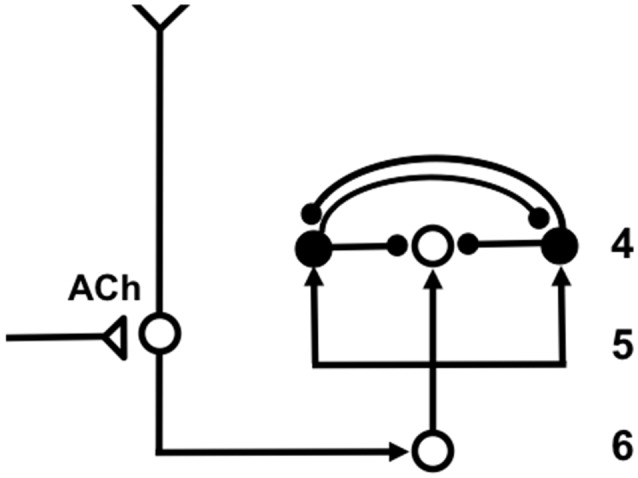
Balanced on-center excitation and total off-surround inhibition are due to self-normalization of total activity by the recurrent off-surround shunting network. This model circuit in cortical layers 4, 5 and 6 is intermittently activated by ACh-modulated bursts from cells in layer 5. The text clarifies how its dynamics capture key properties of Up and Down states during slow wave sleep. Open circles, excitatory cells; closed disks, inhibitory cells.

These recurrent inhibitory circuits naturally lead to properties of Up and Down states during slow wave sleep (Steriade et al., 1993b; Timofeev and Steriade, 1996; Steriade and Timofeev, 2003; Volgushev et al., 2006), including the fast reaction of inhibitory interneurons, and the silencing of both excitatory and inhibitory neurons during the Down state. The simplest version of such a recurrent inhibitory circuit between layers 6 and 4 also includes ACh-modulated inputs from layer 5 cells (see Figure 7D) that intermittently burst during slow wave sleep (Figure 9).

The analysis herein does not explicitly model how layer 5 cells generate intermittent bursts during slow wave sleep as a result of ACh down-regulation, among other factors. Other modeling articles make proposals about how this may happen; e.g., Bazhenov et al. (2002) and Esser et al. (2009). *Given* such a excitatory burst emanating from the layer 5 cell in Figure 9, the burst inputs to both the on-center and the off-surround of the network. If the inhibitory interneurons obey dynamics of fast spiking cells, they may thus begin to be activated a little before the excitatory on-center cells. Despite such a brief advantage, the on-center cells can nonetheless begin to fire before signals from the inhibitory interneurons can take effect at the on-center, due to the extra stage of axonal delay in the off-surround anatomy. This period of on-center activation is an Up state.

This Up state is transient, however, due to the transient nature of the layer 5 excitatory input burst, combined with the balanced total excitatory and inhibitory inputs to the on-center. When the inhibitory interneuronal signals take effect at the on-center cells, the on-center cells will be completely inhibited due to this balance. As the on-center cells are inhibited, so too will the inhibitory interneurons by the recurrent inhibitory feedback that they deliver to each other. When both on-center and off-surround cells are silenced, a Down state is created.

Another relevant factor is that the layer 6-to-4 connections in the LAMINART model are gated by activity-dependent habituative transmitters. These habituating gates help to explain many data, ranging from data about visual persistence (Francis et al., 1994; Francis and Grossberg, 1996) to mismatch-mediated reset (Grossberg, 1976; Grossberg and Versace, 2008). In the case of slow wave sleep, such habituation may influence the duration of the Down state (Sanchez-Vives and Mattia, 2014).

### 11.7 Towards Unified Models of Circadian Rhythms, Sleep, Memory Consolidation and Awake Cognitive-Emotional Behaviors

A great deal more modeling needs to be done to characterize the quantitative properties of the proposed network substrate in Figure 9 of Up and Down states during slow wave sleep. For the present, the most important fact is that this proposal uses the *same* laminar cortical networks that have also been used to quantitatively simulate many data about cortical development, perception, and cognition in awake individuals. This linkage raises new and fascinating questions. For example, the same modulatory on-center, off-surround network in layers 6-to-4 that is used in Figure 9 to qualitatively describe some properties of Up and Down states has also been used to explain and quantitatively simulate many data in awake animals and humans about contrast normalization, attentional modulation, decision-making, activity-dependent habituation, and mismatch-mediated reset, among other properties. It will be instructive in future studies to see if the same network parameters that fit data in the awake state also succeed in providing quantitative fits to data about the durations of Up and Down states during slow wave sleep, and how differences in ACh modulation in the awake and sleep states influence these results.

More modeling also needs to be done to unify neural mechanisms of circadian rhythms and slow wave sleep, and the complex relationships that coordinate these processes (e.g., Achermann et al., 1993; Deboer et al., 2003). Neural models have proposed how the clock-like dynamics of the suprachiasmatic nuclei (SCN) in the hypothalamus are modulated by homeostatic factors like activity-dependent fatigue signals and non-homeostatic factors like the timing of light and dark episodes, to simulate the intensity, duration, and patterning of ultradian activity-rest cycles and the duration of circadian periods under a wide range of light-dark conditions (Carpenter and Grossberg, 1983, 1984, 1985).

This Gated Pacemaker theory of circadian rhythms also suggests how circadian and appetitive hypothalamic circuits may both be constructed from similar components, albeit being specialized to carry out their respective tasks. Indeed, the homeostatic and non-homeostatic inputs in the SCN circuit have homologs in satiety and sensory inputs, respectively, in the appetitive circuits. Within this modeling framework, it can be seen how the SCN model circuits can modulate the sensitivity of the appetitive circuits during the control of a wide range of cognitive-emotional interactions that have also been modeled (e.g., Grossberg, 1972a,b, 1982, 1984b, 2000b; Grossberg and Seidman, 2006; Dranias et al., 2008; Grossberg et al., 2008a).

These cognitive-emotional interactions also include the nSTART model (Figure 4) of the memory consolidation dynamics that occur during sleep and how they are influenced by early or late hippocampal, amygdala, orbitofrontal, or thalamic lesions (Franklin and Grossberg, 2017). It remains to carry out the large-scale modeling program that would be able to unify these emerging strands of theoretical insight that each link brain mechanisms to the psychological functions that they support.

Yet another important linkage that remains to be fully articulated is one between circadian rhythms, sleep, and Alzheimer’s disease, as the next section will discuss. Of particular interest from the present perspective is that major structural degeneration in the brains of Alzheimer’s patients occurs in cortical layers 3 and 5, which are also layers where vigilance control is mediated and where key properties of slow wave sleep seem to be generated (Figure 9).

## 12 ACh, Vigilance, and Alzheimer’s Disease

### 12.1 Alzheimer’s as a Disease of Structurally Impaired Vigilance Control

Alzheimer’s disease includes significant structural abnormalities in many brain regions that may be caused by complex interacting factors. Some of that experimental evidence is reviewed here. In addition, data are reviewed pointing to how this structural degeneration may cause a severe degeneration of cholinergic function in areas of the cerebral cortex, including temporal cortex, that are critical for learning, recognizing, and recalling all sorts of information about the world. Much of the literature focuses on these degenerative structural events. ART clarifies dynamical consequences of these structural events. Indeed, a failure of both tonic and phasic vigilance control could cause a generalized collapse of cognitive processing, way beyond anything that would be caused by the imbalances in the dynamical processing of vigilance that may contribute to behavioral symptoms of autism and medial temporal amnesia.

An early article of Coyle et al. (1983) noted that Alzheimer’s disease was then already one of the most common causes of mental deterioration in the elderly. This article cited compelling evidence that ACh-releasing neurons, whose cell bodies lie in the basal forebrain, selectively degenerate and thereby influence widespread areas of the cerebral cortex and related structures that play an important role in cognitive functions, notably learning and memory. Whitehouse et al. (1982) additionally noted that postmortem studies demonstrated profound reduction in the presynaptic markers for cholinergic neurons in the cortex of patients with Alzheimer’s disease. In particular, neurons of the nucleus basalis of Meynert undergo a greater than 75 percent selective degeneration in these patients. Experimental studies using Alzheimer’s disease animal models have provided supportive data showing that both anticholinergic drugs and lesions of the nucleus basalis of Meynert disrupt learning or memory in a number of paradigms, including passive avoidance learning and Morris water maze tests (Lo Conte et al., 1982; Friedman et al., 1983). Francis et al. (1999), Iqbal and Grundke-Iqbal (2008), and Pimplikar (2009) review these and additional factors that contribute to the disease.

AChE is the main enzyme that can break down ACh in the brain. Inhibition of AChE is thus considered one of the treatment strategies to ameliorate symptoms of Alzheimer’s disease (e.g., Orhan et al., 2004; Mukherjee et al., 2007). Indeed, Janeczek et al. (2017) described an extensive network of cortical pyramidal neurons in the human brain with abundant AChE activity. They quantified the density and staining intensity of these neurons using histochemical procedures. Their methods enabled them to show that brains of adults above age 80 with unusually preserved memory performance (SuperAgers) showed significantly lower staining intensity and density of AChE neurons when compared with same-age peers, leading them to speculate that low levels of AChE activity could enhance the impact of ACh on pyramidal neurons to counterbalance factors that mediate the decline of memory capacity during normal aging.

Rabiei et al. (2014, p. 353) have reported studies that suggest a different approach to enhancing ACh function. They note that Zizyphus jujube (ZJ) activates choline acetyltransferase (ChAT), a transferase enzyme that is responsible for the synthesis of ACh. Their study investigates the effect of ZJ extract in intact rats and in a rat model of Alzheimer’s disease with lesions of the nucleus basalis of Meynert. Learning and memory performance were assessed using a passive avoidance paradigm, and spatial learning and memory were evaluated by the Morris water maze. The results suggested that ZJ has repairing effects on memory and behavioral disorders produced by the nucleus basalis of Meynert lesions, and therefore suggest that ZJ may also have beneficial effects treating Alzheimer’s patients.

Many studies (e.g., Wang et al., 2000a,b) have described the structural degeneration that accompanies and may cause ACh deficits in Alzheimer’s patients, including the role of the 42-amino acid β-amyloid peptide (Aβ_1–42_) in the formation of neuritic plaques and neurodegeneration. Wang et al. (2000a,b) also noted that the α7 nicotinic acetylcholine receptor (α7nAChR) is highly expressed in the basal forebrain cholinergic neurons that project to the hippocampus and cortex of normal and Alzheimer brains (e.g., Perry et al., 1992), correlates well with brain areas that exhibit neuritic plaques in Alzeheimer’s disease, and modulates both calcium homeostasis and release of the neurotransmitter ACh, two important parameters involved in cognition and memory. Nagele et al. (2002) further studied how amyloid β_1–42_ binds with exceptionally high affinity to α7nAChR and accumulates intracellularly in neurons of Alzheimer’s disease brains. Related studies have shown that amyloid peptides can inhibit the release of ACh (Kar et al., 1996).

The above results are consistent with results in Tomlinson et al. (1968, 1970) showing that plaques are more frequent in cortical layers 3 and 5, and in Arnold et al. (1991) summarizing that the laminar distribution of neurofibrillary tangles tended to be selective, involving primarily layers 3 and 5 of association areas; see also Rogers and Morrison (1985). These authors also report far more tangles in both limbic and temporal lobes than in frontal, parietal, and occipital lobes, but also suggested that plaques were more evenly distributed throughout the cortex.

In summary, all of these studies describe the kinds of structural degeneration in layers 3 and 5 that could lead to the massive collapse of ACh function and support of learning and memory that other studies have reported.

### 12.2 Relating Sleep and Alzheimer Disease Pathology

Ju et al. (2014, p. 115) have reviewed evidence that “the sleep-wake cycle directly influences levels of Aβ [amyloid-β peptide] in the brain. In experimental models, sleep deprivation increases the concentration of soluble Aβ and results in chronic accumulation of Aβ, whereas sleep extension has the opposite effect.” The authors go on to note that changes in sleep precede the onset of cognitive symptoms in Alzheimer’s patients, and that disrupted sleep patterns occur in patients with Alzheimer’s disease. Moreover, there is a diurnal variation in the level of soluble Aβ in the interstitial fluid. During the Down state of slow wave sleep, less Aβ is released than during wakefulness or REM sleep. A disruption of slow wave sleep can hereby lead to higher sustained extracellular concentrations of Aβ. These higher sustained Aβ concentrations are, in turn, associated with early amyloid plaque formation.

As noted above, plaques are found more frequently in cortical layers 3 and 5, where they can disrupt the ACh-mediated Up and Down oscillations in slow wave sleep that are diagrammed in Figure 9. By disrupting slow wave sleep in this way, and thereby indirectly causing higher Aβ extracellular concentrations, a vicious cycle can be perpetuated.

### 12.3 Can Novelty-Seeking Behaviors Lower the Chance of Developing Alzheimer’s?

The article’s focus on vigilance control and its review of how normal ACh dynamics are devastated in Alzheimer’s patients raises a question about whether non-invasive behavioral activities that promote normal ACh dynamics, in addition to all appropriate drug and other medical interventions, may help to delay, or lower, the chances of developing Alzheimer’s disease? In other words, can some types of behavior better support the brain’s design to solve the stability-plasticity dilemma (Section 2.1), and thereby allow learning to continue throughout life without a loss of memory stability?

Since unexpected events phasically raise vigilance by releasing ACh (Figure 7), it is natural to ask whether novelty-seeking activities can help to delay, or lower, the chances of developing Alzheimer’s disease? Some data suggest that this may be the case (e.g., Fritsch et al., 2005), while other studies report diminished novelty-seeking behavior in patients with probable Alzheimer’s disease, that is distinct from general cognitive decline (Daffner et al., 1999). It may thus be of use to consider whether and how novelty-rich experiences can be designed to help slow the onset of Alzheimer’s.

## 13 Concluding Remarks

This article discusses the dynamics of tonic and phasic vigilance control within Adaptive Resonance Theory, or ART, laminar cortical circuits. Specializations of these laminar cortical ART models provide a unified explanation, and testable predictions, of many interdisciplinary data about normal and abnormal learning, memory, and behavior. Vigilance in ART is modulated by ACh, often at layer 5 cortical cells that respond to inputs from the nucleus basalis of Meynert. The data that are discussed and explained in this article range from the normal learning and memory of abstract or concrete recognition categories under conditions of low or high vigilance, respectively, and the properties of normal Up and Down states during slow wave sleep, to neural mechanisms and symptoms of mental disorders such as autism, medial temporal amnesia, and Alzheimer’s disease. By mechanistically linking all of these disparate kinds of normal and abnormal data to shared ART network properties, and how they may be modified by different characteristics of tonic or phasic ACh modulation, the article opens the way to designing many new types of experiments that can mechanistically link normal and abnormal behaviors in ways that would be hard to quantify and test without them, and to possible new clinical treatments.

## Author Contributions

The author confirms being the sole contributor of this work and approved it for publication.

## Conflict of Interest Statement

The author declares that the research was conducted in the absence of any commercial or financial relationships that could be construed as a potential conflict of interest.
